# Natural and engineered safe reinforcement learning

**DOI:** 10.1038/s44271-026-00542-4

**Published:** 2026-10-13

**Authors:** Pranav Mahajan, Ben Seymour

**Affiliations:** 1 Oxford Centre for Integrative Neuroimaging, FMRIB, Nuffield Department of Clinical Neurosciences, University of Oxford, Oxford, UK; 2 Max Planck Institute of Biological Cybernetics, Tuebingen, Germany; 3 Institute of Biomedical Engineering, University of Oxford, Oxford, UK

**Keywords:** Human behaviour, Computational science

## Abstract

Survival depends not only on maximising rewards but on minimising harm. While Reinforcement Learning (RL) provides a normative framework for adaptive decision-making, standard algorithms often fail to account for the catastrophic costs of errors during learning, in the same way as animals do. In this review, we synthesise recent advances connecting the engineering of safe RL with behavioural and systems neuroscience. We adopt a principled taxonomy distinguishing between two strategies: (1) modifying the optimisation criterion to embed safety into the learning objective, and (2) modifying the exploration process to prevent dangerous actions. We show that engineering solutions, such as worst-case optimisation, risk-sensitive objectives (e.g., conditional value at risk, CVaR), and constrained Markov decision processes (MDPs), offer precise computational accounts for anxiety, risk-aversion, and defensive reflexes. Conversely, biological mechanisms, including innate priors, interoception, and meta-control based on uncertainty, reliability and competing needs, offer robust solutions to the problem of safe exploration in artificial agents. By bridging these domains, we highlight a converging computational landscape where safety is achieved through a dynamic interplay between reactive instincts and deliberative planning.

## Introduction: the survivor’s dilemma

The notion of ‘learning safely’ may appear paradoxical at first glance. Learning from experience necessarily involves making mistakes, yet some mistakes, such as severe injury or death, can be so catastrophic that they preclude further learning altogether. This raises a fundamental question for any intelligent agent, biological or artificial: how to learn from experience while minimising the risk of potentially irreversible harm?

Prior work suggests that addressing this dilemma requires two seemingly complementary capabilities. The first is *safe exploration*: the ability to act cautiously in uncertain environments to gather information while avoiding external threats. The second is *self-preservation*: the capacity to detect, represent, and respond to deviations from homeostatic grace, i.e., internal threats such as injury in an animal or a component fault in a robot^1^. While safe exploration concerns external dangers, self-preservation focuses on maintaining internal integrity. Both safe exploration and self-preservation are priorities for animals and other intelligent machines wishing to maximise their chances of survival in an uncertain physical world. One way to study these capabilities computationally is through the lens of safe reinforcement learning^2^. Recently, such computational approaches are increasingly relevant to neurobehavioural modelling and human computational psychiatry^3^, particularly in domains involving fear, anxiety, panic, avoidance, aggression, and rumination^4^ as well as in applying principles of computational psychiatry to computers or intelligent machines^5^.

Significant prior work has mapped these computations along the threat-imminence continuum preceding a predatory encounter, varying with the spatial and temporal proximity of danger^6,7^. While acute prey-predator escapes contribute a significant portion of safe behaviours^8^, this review broadens the focus to learning from generic external harms (often treated as losses or punishments). We examine the wider continuum of safe behaviours: from the anticipatory computations underlying risk-aversion and anxiety, to the self-preservative actions that unfold after trauma, such as an injury^9^. By integrating both anticipatory and post-trauma domains, we aim to highlight underexplored areas in computational neuroscience with direct applications to computational psychiatry.

We first introduce the canonical RL framework and how incorporating different forms of innate knowledge can shape the learning of safe behaviours^10^. Building upon the canonical RL framework and the taxonomy of Garcıa and Fernández^2^, we next interpret and categorise common safe reinforcement learning methods as modifications to that canonical framework. In doing so, we highlight how methods modifying the optimisation criterion provide applications to behavioural neuroscience and how behavioural-neuroscience findings provide insights for methods modifying the exploration process. We conclude the review with outstanding questions and future directions enabled by such an interdisciplinary approach.

## Reinforcement learning preliminaries and incorporating innate knowledge

### Learning through reinforcement

Reinforcement learning (RL) provides a mathematical framework for agents to learn how to act in uncertain environments, using feedback from rewards or punishments. At its core, the agent observes a state, takes an action, and receives a reward and a new state from the environment. Over time, the agent aims to learn a policy, a mapping from states to actions, that maximises its cumulative (discounted) reward, also called the *return*^11^.

Formally, these problems are often modelled as Markov decision processes (MDPs), where each action taken in a state leads probabilistically to a new state and a reward (*r*). The *return* from time *t* is typically defined as the discounted sum of future rewards:

1 $${G}_{t}={\sum }_{k=0}^{\infty }{\gamma }^{k}{r}_{t+k+1},$$

where *r*_*t*+*k*+1_ is the reward received upon taking an action *k* timesteps after *t* and *γ* ∈ [0, 1] is a discount factor that shrinks the value of distant rewards^11^. Central to RL is the *value function*, which quantifies the expected return from a state (or state-action pair) under a given policy.

At one end of this spectrum are *model-free* methods, which learn directly from trial and error without constructing an explicit model of the environment. Box 1 provides an introduction to Q-learning, a canonical example of model-free reinforcement learning. At the other end are *model-based* approaches, which build an explicit model of the environment’s transition probabilities, $T(s{\prime} | s,a)$, and reward structure, *R*(*s*, *a*). This model allows the agent to plan by simulating future trajectories, making it more sample-efficient and flexible. The dichotomy between model-free and model-based control parallels the distinction between ‘habitual’ and ‘goal-directed’ behaviours in psychology^12^, where the two systems often compete for behavioural control, with arbitration guided by uncertainty^13^ or reliability^14^.

Many architectures seek a middle ground. *Hybrid approaches*, such as Dyna^15^, integrate both strategies: a learned model generates simulated experiences to train model-free value learning. The Successor Representation offers another compromise, learning predictive relationships between states to capture model-based-like flexibility using simple model-free learning rules^16,17^.

Many of the safe-learning methods discussed later can be understood as a computational modification of the canonical RL framework: modifying either the target inside the TD-error, the transition model that supports it, the objective function being optimised, or the policy used to gather experience etc.

Box 1 Q-learning A canonical example is the Q-learning algorithm^10^, which learns state-action values (Q-values), e.g. *Q*(*s*, *a*), under a greedy target policy (i.e. a policy that takes the action with the highest value at each future time step). After taking action *a*_*t*_ in state *s*_*t*_ and observing reward *r*_*t*+1_ and next state *s*_*t*+1_, the Q-value is updated by:

2 $${\delta }_{t}={r}_{t+1}+\gamma {{\max }_{a^{\prime} }}Q({s}_{t+1},a^{\prime} )-Q({s}_{t},{a}_{t})$$

3 $$Q({s}_{t},{a}_{t})\leftarrow Q({s}_{t},{a}_{t})+\eta {\delta }_{t}$$

4 $$\pi (a| s)=\dfrac{\exp (Q(s,a)/\tau )}{{\sum }_{a^{\prime} }\exp (Q(s,a^{\prime} )/\tau )}$$

where *η* ∈ (0,1] is the learning rate and *δ*_*t*_ is the *temporal-difference (TD) error*. Although Q-learning is defined with a greedy target policy, the behaviour used to sample actions can still be stochastic. For example, a common action-selection rule for modelling animal or human behaviour is the softmax policy: where *τ* > 0 is a temperature parameter: smaller values make action selection more greedy, whereas larger values encourage more exploration. This TD-error is also the prediction-error signal that links these methods to the brain, where related ‘reward prediction errors’ have long been associated with phasic dopamine signals in ventral tegmental area (VTA)^88^ and since then extended to several reward and punishment signals in different brain regions^89,90^.

### Learning through other means and possible forms of innate knowledge

Beyond learning from direct reinforcement, an agent’s behaviour is profoundly shaped by innate structures and prior knowledge. In his foundational thesis,^10^ presciently outlined six categories of innate ‘knowledge’ that constrain and guide learning:

1. Physical capacities (e.g. birds with long beaks etc.)
2. Subjective reward systems
3. Methods of representation and approximation
4. Initial policies, value functions, and models
5. Bounds and constraints on policies, value functions and models
6. Tendencies to experiment (or investigate)

These categories provide a useful scaffold for thinking about safe learning. For instance, innate priors (category 4) act as a first line of defence, shaping behaviour long before instrumental values are learned^18–20^. Similarly, ‘tendencies to experiment’ (category 6) map to information-seeking behaviours crucial for injury management^9,21^. Furthermore, behaviour need not even be individually learned; it can be acquired via vicarious social learning. Observing others’ mistakes is far safer than experiencing consequences oneself, a mechanism supported by neural systems that process observational prediction errors^22^.

Crucially, while these innate constraints ensure baseline survival, their dysregulation provides a powerful framework for computational psychiatry. For example, traditional RL accounts often model consummatory anhedonia simply as a blunted reward function (a failure of category 2). However, recent work by refs. ^23,24^ reconceptualises anhedonia not as a loss of reward, but as a failure to represent and infer progress towards meaningful goals. In Watkins’ taxonomy, this reframes anhedonia as a pathology of representation (category 3), where the perceived distance to a goal is distorted, combined with rigid constraints on the value function (category 5) caused by persisting with unachievable goals. Thus, mapping clinical symptoms to specific innate RL constraints allows us to move beyond simplistic reward-deficit models and identify the precise structural failures driving psychiatric vulnerability.

## An integrated taxonomy of safe learning

While Watkins’ categorisation provides insight into how innate knowledge can be incorporated into the general canonical reinforcement learning framework, and one useful way to think about how biology might implement safe RL, it was not proposed specifically for the categorisation of safe RL.

**Fig. 1:**
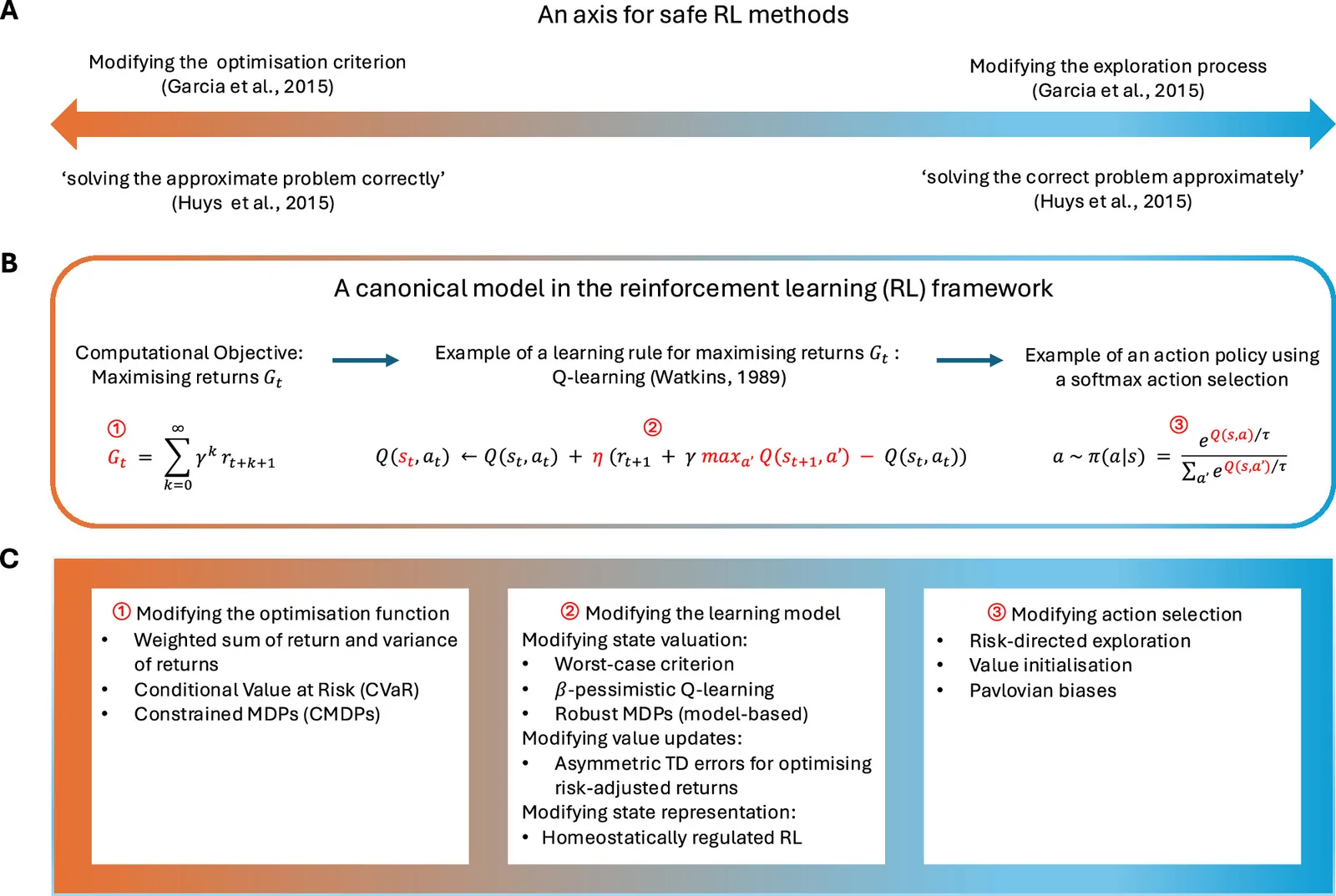
An illustration of different safe RL methods spanning the spectrum from modifying the optimisation criterion to modifying the exploration process^2^. **A** An axis for safe RL methods. On one end, methods modifying the optimisation criterion solve the approximate problem correctly, whereas on the other end, modifying the exploration process leads to an approximate solution to the correct problem. In computational psychiatry, this relates directly to Huys et al. ^3^ categorisation of fault lines in decision-theoretic psychiatry, such as solving the wrong problem and solving the right problem wrongly. **B** A schematic overview of the canonical reinforcement-learning scaffold, highlighting how different methods modify different aspects of the canonical model (or its model-based counterpart): (1) the optimisation function, (2) the learning model, including next-state valuation, value updates, or the state representation, or (3) action selection. Equation components that are often modified are highlighted in red. **C** Major safe RL approaches arranged along the distinction between modifying the optimisation criterion and modifying the exploration process.

Garcıa and Fernández^2^ provide a principled taxonomy for safe RL, dividing methods into two complementary categories, which we present as opposite ends of a spectrum of different safe RL methods (Fig. 1):

- *Modifying the optimisation criterion:* Embedding safety directly into the learning objective (e.g., risk, worst-case outcomes, or constraints).
- *Modifying the exploration process:* Controlling how an agent gathers experience to prevent dangerous actions before they are taken (e.g., priors, demonstrations, or heuristics).

We adopt this dual-category structure to bridge ideas from artificial intelligence and behavioural neuroscience in this review. We argue that while AI has extensively formalised the former category (modifying the optimisation criterion), providing applications to explain different psychiatric phenomena, experimental neuroscience offers rich, evolutionary evidence for the latter. By unpacking how biological agents safely navigate the unknown, we can inspire novel, neuro-inspired approaches for exploration in artificial agents.

At the same time,Garcıa and Fernández's^2^ taxonomy becomes more useful when read together with Watkin's^10^ account of innate knowledge. The same method can therefore be located along at least three dimensions: whether it changes the optimisation criterion or the exploration process; which form of innate knowledge it utilises; and which term of the canonical RL scaffold it changes. This turns the review from a list of proposals into a comparative map (see Table 1 for an overview).

**Table 1:** Integrating Garcıa and Fernández ^2^’s safe-RL taxonomy with Watkins^10^’s taxonomy of innate knowledge

Method	^2^ class	^10^ category	Canonical-update locus	Psychological phenomenon	Practical assumptions and/or limitations
Worst-case and pessimistic value learning^25,26^	Optimisation criterion	3, 5	Replaces optimistic valuation based on future action with a min/max mixture	Anxiety, fear generalisation, low perceived control^28^	Requires specifying how pessimistic future control should be
Robust MDPs^27^	Optimisation criterion	3, 5	Replaces the expectation over a fixed transition model with a worst-case over a model set	Cautious planning under transition model parameter uncertainty	Requires specifying an uncertainty set over dynamics
Variance- or TD-risk objectives^29–31^	Optimisation criterion	2, 3	Modify the quantity being optimised, i.e. counts as valuable return	Risk aversion, asymmetric learning from positive and negative prediction errors^32^	Operationalises risk as the variance in returns; treats uncertainty in positive and negative rewards (punishments) as equally undesirable
Conditional Value at Risk (CVaR)^34,35^	Optimisation criterion	2, 3, 5	Optimises lower-tail outcomes rather than expectation	Timidity and avoidance under rare catastrophe^40,42^	Requires estimating the return distribution or its lower tail
Constrained MDPs^47,48^	Optimisation criterion	2, 5	Adds a constraint on a separate cost return	Behaviour under an explicit upper bound on cumulative costs	Requires explicit, measurable cost signals and a budget
Controllability-based bonuses or modulation of learned reflexes^52^	Exploration process	5, 6	Alters action selection rather than the value itself	Cautious exploration, arbitration under uncertainty^20^	Cheap to compute, but biases experience toward predictable regions, with safer but less efficient behaviours
Priors, demonstrations, and initialisation^66^	Exploration process	4	Shapes starting values, policies, or models	Neophobia^69^, hardwired avoidance behaviour and vicarious learning	Useful early, but may overgeneralise when contexts shift
Interoceptive and self-preservation models	Exploration process with state redesign	2, 3, 6	Addresses origin of rewards, expands state representation to internal states and predictive regulation	Injury, hunger, thirst, sleep management, homeostatic control^21,49,80,81^	Requires internal sensing or inference about latent bodily states to drive optimal behaviour

The table also locates each method on a common RL scaffold and indicates the psychological phenomena it has helped explain.

This integration also helps organise the opening distinction between safe exploration and self-preservation introduced in the Introduction. Some methods primarily protect against external threat; others focus more directly on internal integrity. A recurring theme of the review is that current frameworks rarely handle both jointly in a unified way.

## Modifying the optimisation criterion, with applications to behavioural neuroscience

The first major class of safe RL methods involves altering the learning objective to account for safety. Rather than simply maximising expected return, these approaches embed additional considerations such as risk terms, constraints, or worst-case outcomes directly into the optimisation landscape. In this section, alongside introducing different approaches to modifying the optimisation criterion, we also review their applications to behavioural neuroscience.

### Worst-case criteria

These methods prioritise robustness by maximising performance under the worst outcomes. For example, worst-case $\hat{Q}$-*learning*^25^ optimises over worst-case returns, while *β*-*pessimistic Q-learning*^26^ interpolates between best and worst-case scenarios:

5 $${Q}_{\beta }({s}_{t},{a}_{t})\leftarrow {Q}_{\beta }({s}_{t},{a}_{t})+\eta \left[{r}_{t+1}+\gamma {V}_{\beta }({s}_{t+1})-{Q}_{\beta }({s}_{t},{a}_{t})\right]$$

where the interpolated value function is given by:

6 $${V}_{\beta }({s}_{t+1})=(1-\beta ){{\max }_{a}}Q({s}_{t+1},a)+\beta {{\min }_{a}}Q({s}_{t+1},a)$$

Here, the parameter *β* ∈ [0, 1] serves as a subjective pessimism weight, explicitly quantifying the degree to which an agent anticipates the worst possible future outcome (${{\min }_{a}}Q({s}_{t+1},a)$) versus the best possible future outcome (${{\max }_{a}}Q({s}_{t+1},a)$).

The worst-case principle can also be extended to *model-based* settings to address uncertainty in the transition dynamics. Robust MDPs^27^ assume the true model lies within an uncertainty set ${{\mathcal{P}}}$, where each state-action pair is associated with a set of plausible transition kernels rather than a single known transition model. In practice, the uncertainty set is typically specified by the modeller, though often these sets can be specified from confidence regions around noisy transition estimates or from structured assumptions about model misspecification^27^. The robust value function is then defined as:

7 $${V}^{* }(s)={{\max }_{\pi }}{{\min }_{p\in {{\mathcal{P}}}}}{V}^{\pi ,p}(s)$$

where the agent maximises policy performance under adversarial environment dynamics.

#### Applications to behavioural neuroscience

Worst-case criteria provide a simple yet powerful lens for modelling maladaptive avoidance in anxiety disorders. Zorowitz et al.^28^ use *β*-*pessimistic Q-learning* to formalise how pessimistic beliefs about future control can lead to pathological behaviour. They contrast this with standard Q-learning, where the assumption of optimal future action implies that the potential for future avoidance is protective. In their pessimistic model, a high *β* parameter represents a belief that future avoidance will fail. This single pessimistic assumption leads to an excessive propagation of fear to situations distant from the actual threat, providing a computational account for exaggerated threat appraisals, fear generalisation, and persistent avoidance. This framework further attempts to connect the computational mechanism of pessimism to the development of anxiety and depression, linking it to maladaptive beliefs about self-efficacy and control.

Worst-case methods are related to the risk-sensitive and constrained approaches discussed next, but they are distinguished by what they optimise against: they guard directly against the single worst plausible future, rather than against poor outcomes in the lower tail of a return distribution or violations of a separate cost budget.

### Risk-sensitive criteria

Broadly, there are two ways to incorporate risk-sensitivity in the optimisation criterion: (I) using a weighted sum of return and risk and (II) using conditional value at risk (CVaR).

#### (I) Weighted sum of return and risk

These methods augment the reward-maximising objective with a risk penalty, weighted by a hyperparameter *λ* that balances the trade-off between return and risk:

8 $${{\max }_{\pi }}{{\mathbb{E}}}_{\pi }[G]-\lambda \cdot \omega (G)$$

Here, *G* is the return and *ω*(*G*) denotes a risk measure. *λ* > 0 implies risk-aversion, whereas *λ* < 0 implies risk-seeking preference. Depending on the algorithm, the risk measure *ω*(*G*) can be instantiated as:

- the *variance of returns*^29,30^, penalising uncertain outcomes. Here, the variance of returns term arises from the Taylor expansion of exponential utility functions, to the first order.
- *temporal-difference (TD) errors*^31^. Here, positive and negative prediction errors have asymmetric effects on learning, a concept utilised to design neuroscientific experiments^32^.
- or the *probability of failure or termination in undesirable states*^33^.

##### Applications to behavioural neuroscience:

Risk-sensitive criteria, particularly those based on asymmetric impacts of prediction errors, provide a compelling account of how the brain learns about variance from experience. Niv et al.^32^ tested whether the neural correlates of reinforcement learning are sensitive to risk by having participants learn the values of stimuli with equal means but different variances. They compared a standard TD model, a model with non-linear utility, and a risk-sensitive TD model in which positive and negative prediction errors are weighted by different learning rates. The risk-sensitive model provided the best fit to subjects’ choices, demonstrating that risk aversion correlated with a greater learning rate for negative prediction errors, and vice versa for risk-seeking. Crucially, they showed a close neurometric-psychometric coupling: BOLD signals in the nucleus accumbens, which encoded prediction errors, could be used to extract learned stimulus values. This suggests that risk sensitivity is not just a feature of outcome evaluation but is integral to the learning process itself, implemented via asymmetric updating from positive and negative feedback.

However, defining risk as the variance of returns has a key limitation: it treats positive and negative uncertainty as equally undesirable. A more precise formulation of risk, particularly for safety, should focus specifically on the lower tail of the return distribution, a concept addressed by methods such as conditional value at risk.

#### (II) Conditional value at risk (CVaR)

CVaR^34,35^ targets expected returns under the lower tail of the distribution of returns:

9 $${{\mbox{CVaR}}}_{\zeta }(G)={\mathbb{E}}[G| G\le {v}_{\zeta }(G)]$$

where *v*_*ζ*_(*G*) is the *ζ*-quantile of the return *G*, also known as the value-at-risk. Conceptually, by optimising CVaR of returns, we can formalise an agent’s tendency to focus on the ‘tail events’ or the set of worst-case possibilities, rather than the full spectrum of outcomes. In the limit as *ζ* → 0, CVaR approaches an essentially worst-case criterion, because attention collapses onto the very worst outcomes in the return distribution.

When applied to sequential risk, CVaR comes in two flavours^36^: precommitted CVaR (pCVaR), which precommits to a level of risk at the first choice^37^; and nested CVaR (nCVaR), which reapplies CVaR with the same risk preference at every step in a nested manner^38^.

##### Applications to behavioural neuroscience:

CVaR provides a formal method for modelling risk-sensitive behaviours that prioritise avoiding the worst-case outcomes, a hallmark of anxious phenotypes. Gagne and Dayan^36^ applied a CVaR-based model to a dataset of human choices in the two-step sequential decision task.^39^ They showed many human subjects are highly risk averse in the popular two-step task originally designed to investigate model-based (MB) and model-free (MF) learning and planning. Extending this,^40^ connect CVaR-optimal policies to pathological avoidance behaviours and link risk-sensitive offline planning to the cognitive processes of worry and rumination, applying a model of prioritised replay^41^ with a CVaR objective. In animal behaviour, Shen and Dayan^42^ used a Bayes-adaptive MDP with the nCVaR objective to model individual differences in how mice explore a novel, potentially threatening, object. They showed that the spectrum of behaviour from ‘brave’ to ‘timid’ could be captured by variation in the CVaR risk-sensitivity parameter, *ζ*, along with the animal’s prior beliefs about potential hazards. Together, these studies demonstrate that CVaR is a powerful tool for formally characterising individual differences across a range of risk-sensitive behaviours, from adaptive caution to maladaptive avoidance, in both humans and other animals. Recently, Sui et al.,^43^ formulated decision-making in the Balloon Analogue Risk Task as risk-sensitive exploration in Bayes-adaptive MDPs, with early results showing that pCVaR is more risk-averse than nCVaR in a single trial, for the same nominal risk.

This also clarifies a useful comparison across methods. Worst-case optimisation can be viewed as an extreme form of lower-tail sensitivity. While computing CVaR requires keeping track of the entire return distribution, making it more computationally expensive than the aforementioned methods, it is a plausible solution given recent evidence for distributional RL in dopaminergic neurons in the basal ganglia^44^ and prefrontal cortex^45^, along with more biologically plausible learning algorithms proposed for distributional RL^46^.

### Constrained criteria

Constrained MDPs (CMDPs)^47^ maximise return subject to some expected cost constraints (*c*_*t*_):

10 $${{\max }_{\pi }}{{\mathbb{E}}}_{\pi }\left[{\sum}_{t}{\gamma }^{t}{r}_{t}\right]\quad \,{\mbox{s.t.}}\,\quad {{\mathbb{E}}}_{\pi }\left[{\sum}_{t}{\gamma }^{t}{c}_{t}\right]\le b$$

Used in Constrained Policy Optimisation^48^ and related safety-layer methods, these approaches are especially natural when safety costs should remain separate from ordinary reward.

#### Applications to behavioural neuroscience

While CMDPs are primarily an engineering framework, they offer a useful conceptual lens for understanding how biological agents manage competing objectives. Instead of solving a single, complex constrained optimisation problem, the brain may employ separate, interacting control systems^18,49–51^. For instance, the model proposed by Mahajan et al.,^20^ can be interpreted in this light. In this framework, the instrumental system learns to maximise rewards, while a distinct Pavlovian fear system acts as a dedicated controller to (weakly) minimise constraint violations, such as cumulative pain. This multi-system architecture, where a specialised fear system enforces a soft constraint on the behaviour of a reward-seeking system, represents a neurally plausible mechanism for approximating the solutions to constrained optimisation problems.

This distinction between approaches that collapse rewards and costs into a single objective and those that keep separate cost constraints is likely to matter psychologically. Some harms may be fungible with reward, whereas others, such as severe tissue damage or loss of viability, are better treated as non-negotiable bounds.

#### Synthesis across optimisation criteria

Taken together, worst-case, CVaR, variance-sensitive, and constrained approaches occupy different points on the same design space. They differ in whether they protect against uncertainty in future control, in the environment model, in the lower tail of returns, or in an explicit cost channel. Most primarily address *external* danger, although constrained approaches can also be used to model internal safety budgets such as pain or tissue damage. In practice, these methods are not interchangeable and involve different trade-offs, as highlighted in Table 1: CVaR requires access to a return distribution and can be computationally expensive. Robust MDPs require specification of an uncertainty set, are model-based, and can therefore also be computationally expensive. CMDPs may be less computationally expensive at the implementation level, and may potentially be approximated with learned reflexes; however, they require explicit, measurable cost signals and a dedicated biological pathway for them. Box 2 presents a synthesis of the safety-efficiency trade-off, spanning solutions that modify the optimisation criteria and those modifying the exploration process.

Box 2 The safety-efficiency trade-off in natural and engineered RL A fundamental tension in safe RL exists between minimising harm (safety) and efficient reward accumulation (sample efficiency). This *safety-efficiency trade-off*^20^ represents a special case of the classical exploration-exploitation dilemma: agents, on one hand, can exploit safe actions at the expense of sample efficiency in accruing rewards; or prioritise accruing rewards efficiently, at the cost of accumulating significant punishments or constraint violations. Crucially, this trade-off is contingent on the presence of punishments; without the risk of harm, safety concerns vanish, and the problem reduces to the well-known exploration-exploitation trade-off.

Importantly, recent theoretical work across multiple disciplines, including computer science, supports the notion of such a trade-off across different safe RL methods. While Mahajan et al.,^20^ proposed the safety-efficiency trade-off in the context of incorporating Pavlovian fear biases,^91^ recently demonstrates that in constrained MDPs, stricter safety constraints inevitably degrade the speed of reward learning. Similarly, ref. ^92^ prove a fundamental trade-off between risk sensitivity and sample efficiency. Together, these works suggest the possibility of a fundamental trade-off experienced by biological agents while trying to survive.

Different safe RL strategies navigate this trade-off in distinct ways. Risk-sensitive metrics like CVaR effectively alter the objective function, solving the ‘approximate problem’ with a mathematically ‘correct solution’. In contrast, biological heuristics such as Pavlovian biases maintain the normative survival objective but alter the exploration process by applying an approximate solution, i.e. a hard-wired bias^3^. This distinction also helps frame maladaptive avoidance in computational psychiatry^93^: is the patient optimising a pathological objective, or applying a maladaptive heuristic to a normal objective?

## Modifying the exploration process, with insights from behavioural neuroscience

The class of algorithms modifying the exploration process control how the agent explores during learning, aiming to prevent dangerous actions or states from being visited during learning. In this section, alongside introducing different approaches used by computer scientists to modify the exploration process, we also review how insights from behavioural neuroscience can inform these modifications.

### Risk-directed exploration

A clever approach to safe exploration is to bias the agent towards more predictable, or ‘controllable’, regions of the environment. Gehring and Precup^52^ propose a method to achieve this by defining a state-action pair’s controllability, *C*(*s*, *a*), as the negative of the expected absolute TD-error, $C(s,a)=-{\mathbb{E}}[| {\delta }_{t}| ]$. A running estimate of this score is learned via:

11 $$C({s}_{t},{a}_{t})\leftarrow C({s}_{t},{a}_{t})-{\eta }_{c}(| {\delta }_{t}| +C({s}_{t},{a}_{t}))$$

where *η*_*c*_ is a learning rate for *C*(*s*, *a*). This controllability score is then used as an additive bonus (or as a malus) during action selection, *Q*(*s*, *a*) + *κC*(*s*, *a*). A positive weight *κ* encourages the agent to seek out states with high controllability (i.e., low prediction error variance), effectively biasing exploration towards ‘safer’, more predictable parts of the state space.

#### Insights from behavioural neuroscience

This principle of using measures of controllability to guide safe exploration has a strong parallel in neuroscience. Mahajan et al. ^20^ propose that Pearce-Hall associability^53^ serves as a meta-control signal that arbitrates between instrumental and Pavlovian control. This is conceptually similar to^54^, who also propose a controllability-based arbitration between Pavlovian and instrumental reward systems, except that^20^ focus on the flexible recruitment of the Pavlovian fear system rather than the Pavlovian reward system. Associability is a running average of absolute prediction errors and tracks outcome uncertainty over time, similar to the controllability score from ref. ^52^. However, instead of being an additive bonus to a single controller’s values, Pearce-Hall associability dynamically adjusts the influence of a specialised Pavlovian fear system. High outcome uncertainty increases the weight of the Pavlovian system, enforcing cautious withdrawal behaviours. As the agent learns and uncertainty resolves, control is ceded to the more efficient instrumental system. This represents a neurally plausible mechanism for implementing risk-directed exploration, where uncertainty signals linked to the amygdala and pgACC^55,56^ arbitrate between distinct, specialised controllers. Clinically, a failure to down-regulate this Pavlovian influence once uncertainty resolves may offer one mechanistic explanation for persistent avoidance in general anxiety disorders and in the Fear-Avoidance model of pain chronification^57,58^.

This comparison also brings out a broader point: some safe-exploration methods alter the policy directly, whereas others alter which controller gets to choose the policy. We therefore expect reliability-based arbitration schemes, in which the reliability of different task sets or models of the environment guides which system should control behaviour at a given time^59^, to be as important for safety during decision-making under uncertainty as any single exploration bonus. Woo et al.,^60^ demonstrate how differences in reward prediction errors across competing systems, in their case a stimulus-based and an action-based system, can be used to estimate their relative reliability. They further show that the amygdala adjusts the initial balance between the two learning systems and is essential for updating arbitration according to the correct model, which in turn alters the interaction between arbitration and learning that governs the time course of learning and choice behaviour. We expect such reliability-based methods to be useful in safety-related meta-control, given that they have also previously been used to explain arbitration between model-free and model-based RL in the brain^14^.

Lastly, the general notion of having multiple control systems, each dedicated to different types of reward, has been suggested in computational neuroscience and computational psychology studies^49,50,61,62^. Most relevant to safety are proposals that suggest splitting the controller into two streams, one focused on rewards and the other on punishments^50,51,63^. Having a separate punishment-specific value system ensures that punishment-related fear memories cannot be overwritten by successive rewards, however large they might be, thereby helping safe behaviour via an unaltered fear memory. Wang et al.^64,65^ further extend the model proposed by ref. ^51^ to a deep reinforcement-learning solution for robot navigation, with a state-evaluation-based weighting scheme to determine the mixing weights of reward- and punishment-specific policies under a scaling constraint. They demonstrate that early learning of pain avoidance assisted later goal achievement in experiments with a TurtleBot navigating a variety of mazes with Lidar and image-based inputs. We expect the previously mentioned reliability-based arbitration methods to be potentially useful in further optimising the meta-control between such dual-controller systems.

### Providing initial knowledge

The most elementary method for biasing exploration is to choose an initialisation based on prior knowledge. This is particularly effective for ensuring safety in the early stages of learning, before the agent has gathered enough experience to make accurate value estimates. One common approach is to bootstrap the learning algorithm from a finite set of demonstrations provided by a teacher, termed apprenticeship learning^66^. This biases the agent towards relevant regions of the state-action space from the outset, reducing the time spent in potentially dangerous random exploration. Another way is to meta-learn a value initialisation from a range of environments^67^.

#### Insights from behavioural neuroscience

This engineering principle of safe initialisation has a deep biological analogue in the form of innate priors and evolutionarily shaped value functions. Animals are not born as blank slates; they possess innate behavioural repertoires and value systems that guide their first interactions with the world. For example, neophobia, the fear of novel stimuli, can be understood as a pessimistic value initialisation that promotes cautious avoidance. This aversive value initialisation has been linked to dopamine signals in the tail of the striatum^68–70^. However, a single, fixed pessimistic value initialisation in space cannot explain the dynamic approach-retreat bouts observed in animal exploration^62,71^. Recently propose that behaviour is driven by the dynamic composition of multiple value functions with different initialisations. Gating the influence of these values by an inferred threat-belief state allows the agent to flexibly express innate avoidance, reproducing approach-retreat dynamics and potentially linking these computations to opponent direct and indirect pathways within the tail of the striatum^70^, while also unifying different forms of value-shaping bonuses under a single normative framework^72^. Meanwhile, the tail of the striatum has also been suggested to play a role in shifting attention to orient or avoid^73^.

Lastly, hard-wired safety contributions extend beyond value functions, to state representations and perceptual features signalling threat^74^. McTeague et al.,^75^ show aversive learning shapes neuronal orientation tuning in human visual cortex. More broadly, all of these approaches highlight both the strength and risk of generalisation from priors or approximations. Such generalisation can protect the agent early in learning, but it can also become maladaptive if inherited priors are overgeneralised or fail to update when an environment becomes safer^76^.

### Dedicated internal state and internal models

For physically embodied agents like robots, safety depends not only on the external world but also on the agent’s own internal integrity. This necessitates mechanisms for self-monitoring and fault detection. In control theory and robotics, this is often addressed through model-based fault detection, which involves creating a forward model of the system’s expected dynamics to predict expected sensor readings based on motor commands. A fault is detected when a significant discrepancy, or ‘residual’, emerges between the model’s predictions and the actual sensor readings^77^. A related approach, Associative Skill Memories, learns a generative model of expected sensorimotor observations for different skills, often from demonstration^78,79^. An abnormality or fault can then be detected as a deviation from the learned manifold of expected experience, which can be used to trigger adaptive behaviours.

#### Insights from behavioural neuroscience

This engineering approach has a profound biological parallel in the concept of *interoception*, the brain’s sense of its body’s internal physiological state. The brain maintains sophisticated predictive models of bodily states, a process central to theories of homeostasis and allostasis. Recent interoceptive-AI proposals similarly factorise internal variables from external states, allowing agents to formulate goals based on their own simulated homeostatic needs^80^. This is also a core principle in homeostatically regulated reinforcement-learning frameworks^81^. Mahajan et al.,^21^ treat internal states as partially observable, which can explain costly information-seeking actions (e.g., probing an injury) as rational attempts to reduce uncertainty about the body’s true state to prevent larger future losses. Furthermore, principles of self-supervised predictive coding can be used to learn generative models for interoceptive inference and action^82^. Relatedly,^83^ utilise temporal predictive-coding networks to equip robots with an internal model of expected sensorimotor experiences to detect and react to unexpected events, thereby guiding self-preserving behaviours. This approach is formally related to Kalman filtering^84^, and underscores that safe RL is not only about avoiding external hazards, but also about building and utilising a ‘body model’ of expected embodied sensorimotor observations, analogous to the recently popular ‘world models’ in reinforcement learning^85^. Furthermore, one can consider that major damage, such as a substantial injury, might be incorporated into world and body models rather than the reward function, and as such any cost might be an approximation itself to the long-term estimate of states from which rewards are no longer achievable.

Beyond mere detection, the management of these internal states involves a dynamic arbitration between competing needs. Goldstein et al.,^86^ demonstrate that the need to tend to an injury competes with the need to acquire resources like food or water, resulting in endogenous analgesia where hunger or thirst suppresses sustained pain. They show that these competing drives converge on a specific hub of NPY-receptor-expressing neurons in the parabrachial nucleus, where neuropeptide signalling gates the integration of nociceptive input. This suggests that biological ‘safety’ is not a fixed constraint but a prioritised state, flexibly modulated by the organism’s total homeostatic economy.

#### Synthesis across exploration methods

Priors, controllability bonuses, internal-state models, and arbitration mechanisms all preserve the normative survival objective while changing how behaviour is generated during learning, often termed performance effects and contrasted with learning effects^87^. Compared with optimisation-criterion methods, they are often cheaper and more biologically plausible, but they can also be biased and relatively hard-wired. Many primarily protect against *external* threat, while interoceptive and self-preservative methods more directly address *bodily* integrity, with direct applications to the emerging field of embodied AI.

## Cross-cutting synthesis

Across the reviewed methods, a recurring distinction is whether a proposal changes *what* is optimised or *how* action selection is performed during learning. Methods that alter the optimisation criterion often provide formally transparent solutions, but they can require strong assumptions, such as access to a return distribution, an explicit cost signal, or a well-specified uncertainty set, and can sometimes be computationally expensive, especially if they rely on model-based mechanisms. Methods that alter the exploration process are often computationally cheaper and closer to biological control, but they may rely on heuristics, priors, or controller arbitration that introduce systematic bias.

This distinction also helps reconnect the opening dichotomy between safe exploration and self-preservation. In general, most optimisation-criterion methods are often directed toward external dangers such as threat, punishment, worst-case outcomes, or catastrophic transition dynamics. However, in principle they can be applied to internal/interoceptive threats provided one has a model of an internally generated reward function, e.g., ref.^81^. Exploration-process methods, particularly those involving actions driven by value of information gain, using internal models, and arbitration between competing needs, come closer to explaining how agents preserve bodily integrity once damage or uncertainty about damage has already arisen. However, the boundary between the two is not sharp. For example, an injury tends to heighten an animal’s protective responses, alter mood, and make it generally cautious^9^; this may often be implemented at multiple levels across the spectrum of safe RL methods, from increased risk aversion and rumination to greater reliance on fear biases. However, there is conspicuous scarcity of frameworks that handle both safe exploration and self-preservation jointly, making this a promising direction for future research.

It is also useful to distinguish situations in which unsafe behaviour reflects a failure of safety from those in which it reflects an adaptive decision under pressure. Agents may knowingly accept danger in the face of predictably high costs, for instance by fighting for resources, or impulsive pursuit of rewards. In this sense, biological safe learning is often not the solution of a perfectly specified robust optimisation problem, but a dynamically shifting compromise between safety, reward, control, and computational economy.

## Concluding remarks

In conclusion, this review provides a bridge between the engineering principles of safe reinforcement learning and their analogues in behavioural and systems neuroscience. Computational frameworks from AI provide a precise language for describing *what* an agent should optimise for safety, through concepts like worst-case criteria, risk sensitivity, and constrained MDPs, offering powerful explanatory tools for psychiatric vulnerabilities. Neuroscience, in turn, provides a rich catalogue of mechanisms for *how* agents achieve safety in practice, through innate priors, dedicated internal models, and flexible meta-control. Outstanding questions and future directions are provided in Box 3.

By synthesising these domains, we move closer to both a mechanistic understanding of biological resilience and the design of genuinely robust, self-preserving artificial intelligence. Perhaps the most important open challenge is to build frameworks that can jointly handle external threat, internal integrity, and arbitration between competing needs, while remaining empirically testable in both laboratory tasks and clinical settings.

Box 3 Outstanding questions and future directions While the taxonomy of modifying optimisation criteria and exploration processes provides a robust framework for understanding safe reinforcement learning, several critical frontiers remain unexplored. Further, there is conspicuous scarcity of frameworks that handle both internal and external dangers jointly. Bridging these gaps will require concerted efforts from both algorithmic engineering and systems neuroscience.

• Can we design tasks that distinguish pessimistic beliefs about future control (*β*-pessimistic Q-learning) from lower-tail risk sensitivity (CVaR), rather than collapsing both into a generic form of caution?

• When are harms better treated as explicit constraints rather than as one term in a summed reward-cost objective, and how can this be tested empirically?

• What new benchmarks can computational psychology studies on safe exploration and self-preservation propose for safe embodied AI?

• Can we identify a specific neural representation for the ‘threat-belief state’ and other priorities that potentially gates the influence of innate priors and multiple values in the striatum?

• How can computational frameworks reconcile the opposing defensive drives elicited by threat, where anticipatory anxiety promotes cautious avoidance, while acute danger or tonic pain triggers endogenous analgesia (descending modulation of pain) to facilitate active escape? Building upon this, can one define a unified framework which handles both internal and external dangers jointly?

• How might a biological agent unlearn a once-useful but now obsolete avoidance behaviour when an environment which was previously dangerous becomes safe? Failure to adapt closely relates to the pathology described by ref. ^3^ as the agent solving the problem correctly, but in an unfortunate ‘wrong environment’.

• How do distinct computations responsible for safe reinforcement learning systematically map onto different dimensions of anxiety and trauma-related disorders?

• How can we utilise decision-theoretic models on longitudinal clinical data to develop *behavioural biomarkers* to predict the transitions from acute, adaptive defensive responses to a chronic, dysfunctional state?

## Supplementary information

Transparent Peer Review file

## Data Availability

No data were used in the preparation of any content of this manuscript.
