# Supplementary material for: Natural and engineered safe reinforcement learning
Source: Commun Psychol. 2026 Oct 13;4:138. doi: 10.1038/s44271-026-00542-4 (PMC13661269; doi:10.1038/s44271-026-00542-4)
Supplement: Supplementary file 1 [file 44271_2026_542_MOESM1_ESM.pdf]

nature portfolio

Peer Review File

Natural and Engineered Safe Reinforcement Learning

Corresponding Author: Dr Pranav Mahajan

This file contains all reviewer reports in order by version, followed by all author rebuttals in order by version.

Version 0:

Reviewer comments:

Reviewer #2

(Remarks to the Author)
This is a useful review that brings together proposals from the reinforcement-learning literature and from behavioural and systems neuroscience on how learning can be structured to protect a decision-maker from harm. The topic is timely and well chosen: computational-psychiatry researchers and practitioners will be genuinely interested in it, and the ambition to bridge engineering and neuroscience is exactly the kind of synthesis the field needs. The two-class taxonomy (modifying the optimisation criterion vs. modifying the exploration process) is a sensible organising spine, and the boxes on the safety-efficiency trade-off and on outstanding questions are valuable.

That said, the review does not yet live up to its potential. As written, it largely catalogues individual proposals without explaining them at a level its intended (cross-disciplinary) audience can follow, and without the synthesis that distinguishes a review from an annotated reference list. I am confident the manuscript can become substantially better, and I have tried below to make my suggestions concrete and actionable. Importantly, there is room to do this: Communications Psychology recommends Reviews of up to ~6,000 words with up to 8 display items, whereas the present main text runs to roughly 3,000-3,500 words and uses only 2 of the 8 permitted display items. The expansions I propose can therefore be accommodated comfortably within the journal's limits - indeed, the available space is an argument for making them.

I have three major points.

1. Notation and core algorithms are under-explained for the intended audience

The review explicitly aims to reach readers who are not already fluent in RL, yet the formal machinery is presented as if that fluency could be assumed. Standard symbols (s, a, Q, and so on) are deployed without definition, and several quantities that are doing real work in the equations are never introduced. A reader from clinical or experimental psychology - precisely the reader the authors say they want - will not be able to follow the argument at the points where it matters most.

A few concrete examples of where this currently breaks down:

- The canonical update is never stated. The reader is taken straight to modifications of value learning (Eqs. 1-7) without ever seeing the baseline temporal-difference / Q-learning update that everything else perturbs. The learning rate alpha and discount factor gamma appear in Eq. 1 with no definition; the TD error delta_t is used in Section 4.2.1 and again in Eq. 7

but is never written out or named as the prediction-error signal that links these methods to the brain.

- Symbols are overloaded across equations. This is the most serious source of confusion. The symbol alpha denotes the learning rate in Eq. 1, the tail / confidence level of CVaR in Eq. 5, and is then called the "CVaR risk-sensitivity parameter" in the prose of Section 4.2.2 - three distinct meanings for one glyph. The symbol beta is the pessimism weight in Eqs. 1-2 but the risk-aversion weight in Eq. 4. The symbol omega is a risk-measure functional omega(G) in Eq. 4 but a scalar exploration-bonus weight in Section 5.1 (Q(s,a) + omega*C(s,a)). A non-expert reader has no way to know these are different things; an expert reader is needlessly slowed down.

- Quantities are used before they are defined. The return G appears in Eqs. 4-5 but is only described in passing many paragraphs earlier; the uncertainty set P in the robust-MDP equation (Eq. 3) is named but never characterised (what is in it, who specifies it?); the cost budget d in the constrained-MDP equation (Eq. 6) is left unexplained.

My recommendation is structural and would also do much of the work for point 2 below. I suggest the authors begin from the canonical model-free value update, fully unpacked, e.g.

Q(s,a) <- Q(s,a) + alpha * [r + gamma * max_a' Q(s',a') - Q(s,a)],

defining every term explicitly (state s, action a, action-value Q, reward r, learning rate alpha, discount gamma, and the bracketed term as the TD error delta). Every method that follows can then be introduced as a named modification of one specific term of this update: beta-pessimistic Q-learning replaces the max over future actions with a min/max mixture; robust MDPs replace the expectation over a fixed model with a worst-case over a model set; risk-weighted and CVaR objectives modify the quantity being optimised; CMDPs add a constraint on a separate cost return; the controllability bonus modifies action selection rather than the value itself. Presenting each new parameter at the moment it first modifies the baseline - and stating its range and interpretation - would let a novice reader genuinely follow the logic, and would make the conceptual unity of these methods visible rather than implicit.

2. The two taxonomies are never integrated

Section 2 introduces Watkins' six categories of innate "knowledge," and Section 3 introduces Garcia et al.'s two-class taxonomy (optimisation criterion vs. exploration process). Both are helpful - but they are presented in isolation, and the reader is left to wonder throughout Sections 4-5 how each algorithm relates back to the six-point scheme. I found myself repeatedly trying to do this mapping in my head; the review should do it for me.

I strongly recommend the authors make the relationship between the two taxonomies explicit and carry it through the whole review. The cleanest way to do this is a single integrating display item (the authors have the budget for it) - a table in which each method is a row, with columns for: (i) the Garcia class it belongs to; (ii) the Watkins category(ies) it instantiates; (iii) which term of the canonical update it modifies (per point 1); and (iv) the psychiatric phenomenon it has been used to model. Even a skeleton along the following lines would transform the reader's ability to navigate the material.

3. Sections 4 and 5 list proposals but do not synthesise them

This is the deepest issue. The two core sections give a reasonable overview of what various authors have proposed, but a review's value lies in synthesis - an integrated account of how the pieces relate, what is shared, what is genuinely distinct, and what is missing - rather than in a sequential summary. At present these sections read closer to an annotated lookup table than to an argument. Several specific, achievable additions would fix this:

- Make the formal relationships among the methods explicit. Right now the section-4 methods sit in silos, but they form a connected family. Worst-case

optimisation (Eqs. 2-3) is the limiting case of CVaR (Eq. 5) as the tail level alpha -> 0; beta-pessimistic Q-learning at beta = 1 reduces to Heger's worst-case Q-learning; variance-penalised objectives differ from CVaR precisely in penalising upside and downside symmetrically rather than focusing on the lower tail. Stating these relationships converts a list into a structured map and would help the reader understand why one would choose one method over another.

- Use the safety-efficiency trade-off as an organising axis, not just a box. Box 1 contains what is arguably the central insight of the whole review - that optimisation-criterion methods "solve the wrong problem correctly," while biological/exploration heuristics "solve the right problem approximately." This distinction deserves to be the spine of Sections 4-5 rather than sidebar material: each method could be positioned explicitly on the trade-off, with a brief statement of what it sacrifices (e.g., CVaR alters the objective; Pavlovian biases preserve the objective but distort the policy).

- Reconnect to the review's own opening dichotomy. The Introduction defines two complementary capabilities - safe exploration (external threats) and self-preservation (internal/interoceptive threats) - but Sections 4-5 never map the surveyed methods back onto that split, and the two capabilities are not reunited anywhere. A short synthesis noting which methods address external vs. internal danger (and the conspicuous scarcity of frameworks that handle both jointly) would be a strong, original contribution and a natural bridge to the open questions in Box 2.

- Compare utility and applicability. A reader wants guidance on when each approach is appropriate. A few sentences (or an added column in the table above) on the practical demands and assumptions of each method - CVaR requires estimating the return distribution; CMDPs require an explicit, measurable cost signal; Pavlovian/associability heuristics are cheap but introduce systematic bias; robust MDPs require specifying an uncertainty set - would give the review the comparative, evaluative character that readers will actually use.

Minor points

- A schematic figure would help enormously and the authors have the display-item budget for one: the canonical update with the term modified by each method highlighted would make the entire conceptual structure legible at a glance to the non-expert audience. The review currently contains no figures.

- Define the return G, the TD error delta, the discount gamma, and the learning rate alpha once, early, in a clearly demarcated "preliminaries" passage, and reuse them consistently.

- Consider disambiguating the CVaR tail level from the learning rate notationally (or at least flagging the clash in text), since both are conventionally written alpha.

- The self-citation density is high in places (e.g., the safety-efficiency trade-off and several mechanism mappings rest heavily on the authors' own work); a slightly broader framing where alternatives exist would strengthen the review's authority.

I want to be clear that I am positive about this manuscript and about its prospects. The topic is important, the framing is promising, and the authors are evidently well placed to write the definitive short review on it. My recommendations are aimed at helping them realise that potential: explain the formal machinery so a non-specialist can follow it; integrate the two taxonomies explicitly and carry that integration through; and replace the sequential listing in Sections 4-5 with genuine synthesis. The journal's length and display-item allowances leave ample room for all of this. With these revisions the review would be considerably more useful - and considerably more cited - than in its present form.

Reviewer #4

(Remarks to the Author)

Reviewer #5

(Remarks to the Author)

Version 1:

Reviewer comments:

Reviewer #2

(Remarks to the Author)
The authors have been very receptive to my feedback and have by and large followed my recommendations for improving the accessibility of their review paper. I think the target audience will now be able to learn a lot from reading this review, and I expect that this paper will be very useful for, and widely cited by, clinical researchers wanting to work with these model classes.

Reviewer #5

(Remarks to the Author)
The authors have adequately addressed my concerns. I have no further comments.

Open Access This Peer Review File is licensed under a Creative Commons Attribution 4.0 International License, which permits use, sharing, adaptation, distribution and reproduction in any medium or format, as long as you give appropriate credit to the original author(s) and the source, provide a link to the Creative Commons license, and indicate if changes were made.
In cases where reviewers are anonymous, credit should be given to 'Anonymous Referee' and the source.
The images or other third party material in this Peer Review File are included in the article's Creative Commons license, unless indicated otherwise in a credit line to the material. If material is not included in the article's Creative Commons license and your intended use is not permitted by statutory regulation or exceeds the permitted use, you will need to obtain permission directly from the copyright holder.
To view a copy of this license, visit https://creativecommons.org/licenses/by/4.0/

Response to the Reviewers

Natural and Engineered Safe Reinforcement Learning

Colour key
Blue boxes repeat reviewers' comments.
Green boxes provide our point-by-point response.

Reviewer #2

Reviewer #2 opening assessment

"This is a useful review that brings together proposals from the reinforcement-learning literature and from behavioural and systems neuroscience on how learning can be structured to protect a decision-maker from harm. The topic is timely and well chosen: computational-psychiatry researchers and practitioners will be genuinely interested in it, and the ambition to bridge engineering and neuroscience is exactly the kind of synthesis the field needs. The two-class taxonomy (modifying the optimisation criterion vs. modifying the exploration process) is a sensible organising spine, and the boxes on the safety-efficiency trade-off and on outstanding questions are valuable."

Response

We thank the reviewer for this encouraging assessment and for the concrete suggestions on how to make the review more useful to a cross-disciplinary readership.

Reviewer #2 framing assessment

"That said, the review does not yet live up to its potential. As written, it largely catalogues individual proposals without explaining them at a level its intended (cross-disciplinary) audience can follow, and without the synthesis that distinguishes a review from an annotated reference list. I am confident the manuscript can become substantially better, and I have tried below to make my suggestions concrete and actionable. Importantly, there is room to do this: Communications Psychology recommends Reviews of up to ~6,000 words with up to 8 display items, whereas the present main text runs to roughly 3,000-3,500 words and uses only 2 of the 8 permitted display items. The expansions I propose can therefore be accommodated comfortably within the journal's limits - indeed, the available space is an argument for making them.
I have three major points."

Response

Thank you and we appreciated this framing and revised the paper. The manuscript is now longer, more pedagogical, and more explicitly synthetic, and it makes fuller use of the available display-item budget through Figure 1, Table 1, Box 1, Box 2 and Box-3. The word count of the revised paper is 4902 words.

Reviewer #2, Point 1

“1. Notation and core algorithms are under-explained for the intended audience

The review explicitly aims to reach readers who are not already fluent in RL, yet the formal machinery is presented as if that fluency could be assumed. Standard symbols (s, a, Q, and so on) are deployed without definition, and several quantities that are doing real work in the equations are never introduced. A reader from clinical or experimental psychology - precisely the reader the authors say they want - will not be able to follow the argument at the points where it matters most.

A few concrete examples of where this currently breaks down:

- The canonical update is never stated. The reader is taken straight to modifications of value learning (Eqs. 1-7) without ever seeing the baseline temporal-difference / Q-learning update that everything else perturbs. The learning rate alpha and discount factor gamma appear in Eq. 1 with no definition; the TD error delta_t is used in Section 4.2.1 and again in Eq. 7 but is never written out or named as the prediction-error signal that links these methods to the brain.
- Symbols are overloaded across equations. This is the most serious source of confusion. The symbol alpha denotes the learning rate in Eq. 1, the tail / confidence level of CVaR in Eq. 5, and is then called the "CVaR risk-sensitivity parameter" in the prose of Section 4.2.2 - three distinct meanings for one glyph. The symbol beta is the pessimism weight in Eqs. 1-2 but the risk-aversion weight in Eq. 4. The symbol omega is a risk-measure functional omega(G) in Eq. 4 but a scalar exploration-bonus weight in Section 5.1 (Q(s,a) + omega*C(s,a)). A non-expert reader has no way to know these are different things; an expert reader is needlessly slowed down.
- Quantities are used before they are defined. The return G appears in Eqs. 4-5 but is only described in passing many paragraphs earlier; the uncertainty set P in the robust-MDP equation (Eq. 3) is named but never characterised (what is in it, who specifies it?); the cost budget d in the constrained-MDP equation (Eq. 6) is left unexplained.

My recommendation is structural and would also do much of the work for point 2 below. I suggest the authors begin from the canonical model-free value update, fully unpacked, e.g.

Q(s,a) <- Q(s,a) + alpha * [r + gamma * max_a’ Q(s’,a’) - Q(s,a)],

defining every term explicitly (state s, action a, action-value Q, reward r, learning rate alpha, discount gamma, and the bracketed term as the TD error delta). Every method that follows can then be introduced as a named modification of one specific term of this update: beta-pessimistic Q-learning replaces the max over future actions with a min/max mixture; robust MDPs replace the expectation over a fixed model with a worst-case over a model set; risk-weighted and CVaR objectives modify the quantity being optimised; CMDPs add a constraint

on a separate cost return; the controllability bonus modifies action selection rather than the value itself. Presenting each new parameter at the moment it first modifies the baseline - and stating its range and interpretation - would let a novice reader genuinely follow the logic, and would make the conceptual unity of these methods visible rather than implicit."

Response to Point 1

We agree and substantially revised the exposition. The new section *Reinforcement Learning Preliminaries and Incorporating Innate Knowledge* now introduces the canonical RL setup and the return; the Q-learning update, the TD error and an example softmax action-selection rule in Box-1 (as well as Fig 1(B)) before any modified algorithms are discussed. We also standardised notation across the paper: the learning rate is denoted by η, the pessimism parameter by β, the generic risk weight by λ, the CVaR tail parameter by ζ, the softmax temperature by τ, and the exploration-bonus weight by κ. We now also state more clearly that the robust-MDP uncertainty set is typically specified by the modeller, often informed by confidence regions or model-misspecification assumptions, and that the CMDP cost budget is an externally specified bound. Thank you very much!

Reviewer #2, Point 2

"2. The two taxonomies are never integrated
Section 2 introduces Watkins' six categories of innate "knowledge," and Section 3 introduces Garcia et al.'s two-class taxonomy (optimisation criterion vs. exploration process). Both are helpful - but they are presented in isolation, and the reader is left to wonder throughout Sections 4-5 how each algorithm relates back to the six-point scheme. I found myself repeatedly trying to do this mapping in my head; the review should do it for me.
I strongly recommend the authors make the relationship between the two taxonomies explicit and carry it through the whole review. The cleanest way to do this is a single integrating display item (the authors have the budget for it) - a table in which each method is a row, with columns for: (i) the Garcia class it belongs to; (ii) the Watkins category(ies) it instantiates; (iii) which term of the canonical update it modifies (per point 1); and (iv) the psychiatric phenomenon it has been used to model. Even a skeleton along the following lines would transform the reader's ability to navigate the material."

Response to Point 2

We have now addressed this directly in the section *An Integrated Taxonomy of Safe Learning.* Table 1 now maps each major method onto the Garcia and Fernandez class, the relevant Watkins category, the locus of modification in the canonical RL scaffold, the associated psychological phenomenon, and the main practical assumptions or limitations as suggested by Reviewer #2. Figure 1 was added as a companion schematic to help orient readers before they reach the detailed sections. Thank you very much!

Reviewer #2, Point 3

"3. Sections 4 and 5 list proposals but do not synthesise them
This is the deepest issue. The two core sections give a reasonable overview of what various authors have proposed, but a review's value lies in synthesis - an integrated account of how the pieces relate, what is shared, what is genuinely distinct, and what is missing - rather than in a sequential summary. At present these sections read closer to an annotated lookup table than to an argument. Several specific, achievable additions would fix this:
- Make the formal relationships among the methods explicit. Right now the section-4 methods sit in silos, but they form a connected family. Worst-case optimisation (Eqs. 2-3) is the limiting case of CVaR (Eq. 5) as the tail level alpha -> 0; beta-pessimistic Q-learning at beta = 1 reduces to Heger's worst-case Q-learning; variance-penalised objectives differ from CVaR precisely in penalising upside and downside symmetrically rather than focusing on the lower tail. Stating these relationships converts a list into a structured map and would help the reader understand why one would choose one method over another.
- Use the safety-efficiency trade-off as an organising axis, not just a box. Box 1 contains what is arguably the central insight of the whole review - that optimisation-criterion methods "solve the wrong problem correctly," while biological/exploration heuristics "solve the right problem approximately." This distinction deserves to be the spine of Sections 4-5 rather than sidebar material: each method could be positioned explicitly on the trade-off, with a brief statement of what it sacrifices (e.g., CVaR alters the objective; Pavlovian biases preserve the objective but distort the policy).
- Reconnect to the review's own opening dichotomy. The Introduction defines two complementary capabilities - safe exploration (external threats) and self-preservation (internal/interoceptive threats) - but Sections 4-5 never map the surveyed methods back onto that split, and the two capabilities are not reunited anywhere. A short synthesis noting which methods address external vs. internal danger (and the conspicuous scarcity of frameworks that handle both jointly) would be a strong, original contribution and a natural bridge to the open questions in Box 2.
- Compare utility and applicability. A reader wants guidance on when each approach is appropriate. A few sentences (or an added column in the table above) on the practical demands and assumptions of each method - CVaR requires estimating the return distribution; CMDPs require an explicit, measurable cost signal; Pavlovian/associability heuristics are cheap but introduce systematic bias; robust MDPs require specifying an uncertainty set - would give the review the comparative, evaluative character that readers will actually use."

Response to Point 3

We strengthened synthesis throughout the core sections. We make the formal relationships among methods explicit, as suggested by the reviewer. We make the insights buried in previous Box 1, more explicit and highlight the organising axis in the Fig 1 and thank for the reviewer for pointing this out as the central insight of the whole review. Across multiple sections, we attempt to reunite the review with the opening dichotomy (safe exploration and self-preservation) and also introduce a new section called Cross-cutting Synthesis with noting which methods address

external vs. internal danger (and the conspicuous scarcity of frameworks that handle both jointly). Lastly, practical assumptions and trade-offs are also now made explicit in Table 1 and in the synthesis across optimisation criteria. We also significantly change some outstanding questions in Box 3.

Reviewer #2 minor points

"Minor points
- A schematic figure would help enormously and the authors have the display-item budget for one: the canonical update with the term modified by each method highlighted would make the entire conceptual structure legible at a glance to the non-expert audience. The review currently contains no figures.
- Define the return G, the TD error delta, the discount gamma, and the learning rate alpha once, early, in a clearly demarcated "preliminaries" passage, and reuse them consistently.
- Consider disambiguating the CVaR tail level from the learning rate notationally (or at least flagging the clash in text), since both are conventionally written alpha.
- The self-citation density is high in places (e.g., the safety-efficiency trade-off and several mechanism mappings rest heavily on the authors' own work); a slightly broader framing where alternatives exist would strengthen the review's authority."

Response to Minor Points

We addressed these directly. Figure 1 now provides a schematic overview of the scaffold and an organising axis for safe-RL as suggested by you in an earlier comment. The preliminaries section and new Box 1 defines the return, discount factor, TD error, and canonical update early in the manuscript. Notation has been disambiguated throughout, especially for the learning rate, CVaR tail parameter, and softmax temperature. We also broadened the framing in multiple places by adding neighbouring work on reliability-based arbitration from Soltani group, dual-controller systems from Cohen group, a new example of implementing neuro-inspired reward and punishment-based deep RL on safe exploring Turtlebot by Wang et al. (2018, 2021), more references on CVaR, more references on hard-wired safety systems in the perceptual system, interoceptive control, safety-related representations and more.

Reviewer #2 closing assessment

"I want to be clear that I am positive about this manuscript and about its prospects. The topic is important, the framing is promising, and the authors are evidently well placed to write the definitive short review on it. My recommendations are aimed at helping them realise that potential: explain the formal machinery so a non-specialist can follow it; integrate the two taxonomies explicitly and carry that integration through; and replace the sequential listing in Sections 4-5 with genuine synthesis. The journal's length and display-item allowances leave ample room for all of this. With these revisions the review would be considerably more useful - and considerably more cited - than in its present form."

Response

We are incredibly grateful for this constructive closing assessment and revised the paper these recommendations into account. We believe the paper is greatly improved, thanks to your recommendations. Thank you!

Reviewer #4

Reviewer #4 opening assessment

"Thank you for sending me this paper by Mahajan and Seymour for review. In it, they outline the key issues behind reinforcement learning in the presence of danger, and cogently list the core approaches. They divide the approaches into two broad sets of algorithms - those that modify the criterion, and those that modify the exploration strategy. The paper is superbly clear, and lays out the key approaches cogently and succinctly. For each approach, they state the core mathematical definition, briefly explain the rationale, and then relate it to neuroscience. The paper is very well written and makes an important, clear contribution to the literature.
I only have a few thoughts, but appreciate fully that addressing all of these will not be feasible within the constraints of this brief article, so I mention these more as possibilities to consider exploring (safely, without the threat of rejection)."

Response

We thank the reviewer for this generous assessment and for their suggestions.

Reviewer #4, First Point

"First, it would be nice to more clearly state and outline why and when, formally, separate consideration of costs and rewards is better or worse than their joint consideration as a simple sum. Furthermore, one can consider that major damage might enter more in the world model than the reward function, and as such any cost might be an approximation itself to the long-term estimate of states from which rewards are no longer achievable."

Response to First Point

We expanded the CMDP section and the synthesis across optimisation criteria to address this directly. The revised manuscript now distinguishes approaches that collapse rewards and costs into a single objective from those that preserve separate cost bounds, and explicitly notes that some harms are better treated as non-negotiable limits or threats to future viability rather than as ordinary penalties. We further extend the existing content on internal models/self-preservation to a concept of 'body models' analogous to the 'world models' in reinforcement learning (Ha and Schmidhuber, 2018). We agree, that one may consider that major damage (e.g. a substantial injury) might be incorporated in the world and body model than the reward function and have mentioned it in the Dedicated internal state and internal model section.

Thank you very much!

Reviewer #4, Second Point

"Second, it might be worthwhile to consider not only safe, but also unsafe behaviours, e.g. the face of predictably high costs - for instance fights for resources, or impulsive pursuit of rewards."

Response to Second Point

We now discuss this explicitly in the cross-cutting synthesis, where we distinguish failures of safety from adaptive risk acceptance under pressure. Related themes also now appear in Box 3, especially in the outstanding questions about endogenous analgesia, threat-reward conflicts, and the transition from adaptive defence to dysfunction.

Reviewer #4, Third Point

"Third, it seems that it would be helpful to consider opportunity costs or their opposite, or more broadly the challenge of costly computation."

Response to Third Point

We now discuss this in the subsections summarising the synthesis on modification of the optimisation criterion, exploration process and the cross-cutting synthesis section. We compare multiple approaches and highlight their trade-off in terms of cost of computation and what are biologically plausible. The revised manuscript now frames some biological safety heuristics as computationally cheaper approximations, and it partly discusses safety as a compromise between harm minimisation, reward, control, and computational economy.

Reviewer #4, Fourth Point

"Fourth, might it be also worth be worth considering (safe or unsafe) generalization whereby unknown values are initiated in based on approximations?"

Response to Fourth Point

We expanded this in the subsection on priors, demonstrations, and initialisation. The revised text now treats inherited priors and generalisation as both a source of early safety and a possible source of maladaptive overgeneralisation when environments change.

Reviewer #4, Final Point

"The final thought is that it would be fantastic to have slightly more specific future directions

in terms of how to disentangle the possibilities empirically."

Response to Final Point

We revised Box 3 accordingly. The future-directions section now contains more discriminative questions about pessimism versus tail-risk sensitivity, explicit constraints versus summed objectives etc. Thank you very much for your suggestions, which significantly improved our review.

Reviewer #5

Reviewer #5 opening assessment

"This is an interesting review that attempts to bridge ideas from computer science and neuroscience to develop a framework for safe reinforcement learning (RL). Overall, the manuscript is well written, clear, and provides a balanced overview of the relevant literature. I have only a few comments:"

Response

We thank the reviewer for this positive assessment and for the helpful suggestions on how to strengthen the bridge between neuroscience and RL.

Reviewer #5, Point 1

"1. The overall goal and approach of the manuscript are compelling. However, the integration of the neuroscience and RL perspectives could be developed further. At present, the two strands are discussed largely in parallel, without being connected in a specific or mechanistic way. It would strengthen the paper if the authors could provide one or two concrete examples illustrating how insights from neuroscience and psychology can inform or be incorporated into RL frameworks for safe decision-making."

Response to Point 1

We strengthened this integration throughout the paper. In particular, the section on risk-directed exploration now connects controllability and reliability with arbitration between controllers, while the section on dedicated internal state and internal models more explicitly links interoception, body models/world models, injury-state inference, and self-preserving control. Figure 1 was also designed to make these mechanistic bridges more visible. Importantly, we now provide a new example of implementing neuro-inspired reward and punishment-based deep RL on safe exploring Turtlebot by Wang et al. (2018, 2021), with more possibilities for embodied AI. Thank you!

Reviewer #5, Point 2

"2. In Section 5 (starting around line 174), the discussion is particularly interesting. I wonder whether a related quantity—the difference in reward prediction errors across competing systems, which can be used to estimate their relative reliability (see Woo et al., Nature Communications, 2025)—might be relevant to safe RL. More generally, estimates of the reliability of different task sets or models of the environment can guide which system should control behavior at a given time (e.g., Soltani & Koechlin, 2022). Such reliability-based arbitration mechanisms may provide a useful framework for determining safe actions under uncertainty."

Response to Point 2

We found this suggestion very helpful and integrated it into the section on risk-directed exploration. The revised manuscript now explicitly discusses reliability-based arbitration in detail, including the use of differences in prediction errors across systems, the contribution from amygdala, and relates this both to safety-related meta-control and to earlier work on reliability-based arbitration between model-based versus model-free RL in the brain. Thank you!

Reviewer #5, Point 3

"3. Some abbreviations are not defined at first use (e.g., MDP, CVaR). Defining these terms when they first appear would improve accessibility for a broader audience."

Response to Point 3

We corrected this throughout. MDPs, return, TD error, softmax action selection, and CVaR are now defined in the preliminaries section or at first local use and in Box 1, and notation is now more consistent across the manuscript. Thank you!

Closing Note

Closing note

We are incredibly grateful to the editor and reviewers for thoughtful and constructive feedback. Their comments led us to revise not only the exposition but also the manuscript's overall structure, synthesis, and empirical framing. We believe the revised version is substantially stronger as a result. Thank you very much.
